# Verification of acromion marker cluster and scapula spinal marker cluster methods for tracking shoulder kinematics: a comparative study with upright four-dimensional computed tomography

**DOI:** 10.1186/s12891-024-07717-2

**Published:** 2024-07-26

**Authors:** Yuki Yoshida, Noboru Matsumura, Yoshitake Yamada, Azusa Miyamoto, Satoshi Oki, Minoru Yamada, Yoichi Yokoyama, Masaya Nakamura, Takeo Nagura, Masahiro Jinzaki

**Affiliations:** 1 Department of Orthopedic Surgery, Keio University School of Medicine, 35 Shinanomachi, Shinjuku-ku, Tokyo, 160-8582 Japan; 2 Department of Radiology, Keio University School of Medicine, 35 Shinanomachi, Shinjuku-ku, Tokyo, 160-8582 Japan; 3 Department of Orthopedic Surgery, Fussa Hospital, 1-6-1 Kamidaira, Fussa, 197-8511 Tokyo Japan; 4 Keiyu Orthopaedic Hospital, 2267, Akoda, Tatebayashi, 374-0013 Gumma Japan

**Keywords:** Attachment position, Scapula spine, Biomechanics, Shoulder motion, Scapular tracking, Motion capture, Upright computed tomography

## Abstract

**Background:**

This study validated the accuracy of the acromion marker cluster (AMC) and scapula spinal marker cluster (SSMC) methods compared with upright four-dimensional computed tomography (4DCT) analysis.

**Methods:**

Sixteen shoulders of eight healthy males underwent AMC and SSMC assessments. Active shoulder elevation was tracked using upright 4DCT and optical motion capture system. The scapulothoracic and glenohumeral rotation angles calculated from AMC and SSMC were compared with 4DCT. Additionally, the motion of these marker clusters on the skin with shoulder elevation was evaluated.

**Results:**

The average differences between AMC and 4DCT during 10°−140° of humerothoracic elevation were − 2.2° ± 7.5° in scapulothoracic upward rotation, 14.0° ± 7.4° in internal rotation, 6.5° ± 7.5° in posterior tilting, 3.7° ± 8.1° in glenohumeral elevation, − 8.3° ± 10.7° in external rotation, and − 8.6° ± 8.9° in anterior plane of elevation. The difference between AMC and 4DCT was significant at 120° of humerothoracic elevation in scapulothoracic upward rotation, 50° in internal rotation, 90° in posterior tilting, 120° in glenohumeral elevation, 100° in external rotation, and 100° in anterior plane of elevation. However, the average differences between SSMC and 4DCT were − 7.5 ± 7.7° in scapulothoracic upward rotation, 2.0° ± 7.0° in internal rotation, 2.3° ± 7.2° in posterior tilting, 8.8° ± 7.9° in glenohumeral elevation, 2.0° ± 9.1° in external rotation, and 1.9° ± 10.1° in anterior plane of elevation. The difference between SSMC and 4DCT was significant at 50° of humerothoracic elevation in scapulothoracic upward rotation and 60° in glenohumeral elevation, with no significant differences observed in other rotations. Skin motion was significantly smaller in AMC (28.7 ± 4.0 mm) than SSMC (38.6 ± 5.8 mm). Although there was smaller skin motion in AMC, SSMC exhibited smaller differences in scapulothoracic internal rotation, posterior tilting, glenohumeral external rotation, and anterior plane of elevation compared to 4DCT.

**Conclusion:**

This study demonstrates that AMC is more accurate for assessing scapulothoracic upward rotation and glenohumeral elevation, while SSMC is preferable for evaluating scapulothoracic internal rotation, posterior tilting, glenohumeral external rotation, and anterior plane of elevation, with smaller differences compared to 4DCT.

## Background

Optical motion capture (OMC) systems are widely used to evaluate scapular kinematics in a non-invasive manner. However, soft-tissue artifact (STA) of the scapula is larger than that of other bones because the scapula is deeply located and glides beneath the skin surface, slipping between the skin and bone [1]. For accurate analysis, it is necessary to compensate for scapular STA.

The acromion marker cluster (AMC) method [2] which involves the attachment of marker clusters on the acromion is one of the most common methods to compensate for scapular STA. Even with this method, scapular STA is larger with high shoulder elevations, and it can be accurately assessed up to 90° of elevation [3].

Although scapular skin marker movement is smallest on the acromion [4, 5], the AMC is sensitive to deltoid muscle contraction [6]. As the scapular spine is thought to be minimally affected by deltoid muscle contraction and unaffected by other superficial muscles, a study employed a scapular spinal marker cluster (SSMC) to track scapular motion [7]. However, the accuracy of the SSMC method during active shoulder elevation is unclear.

Recently, we have developed upright computed tomography (CT) [8], which enables the acquisition of whole-body images in a standing position. This machine can be used to evaluate the dynamic motion of the shoulder girdle during active elevation through four-dimensional (4D) scanning. It allows the dynamic verification of OMC during active shoulder motion [9] and is not a static verification method such as palpation [10, 11].

This study aimed to compare the accuracy of scapular motion tracking during shoulder elevation between AMC and SSMC using upright 4DCT. We hypothesized that SSMC would be more accurate than AMC, without the influence of deltoid muscle contraction.

## Methods

### Participants

Eight healthy male volunteers (age, 21.8 ± 1.7 years; height 167.5 ± 3.7 cm; body weight 63.3 ± 3.7 kg; and body mass index 22.6 ± 1.3 kg/m^2^), with no history of shoulder pathology, were included in the study. All participants were right-handed and underwent assessments of both the shoulders. This study was approved by our Institutional Review Board (study protocol: #20,150,293), and informed consent was obtained from each participant.

### Data collection device

**Fig. 1:**
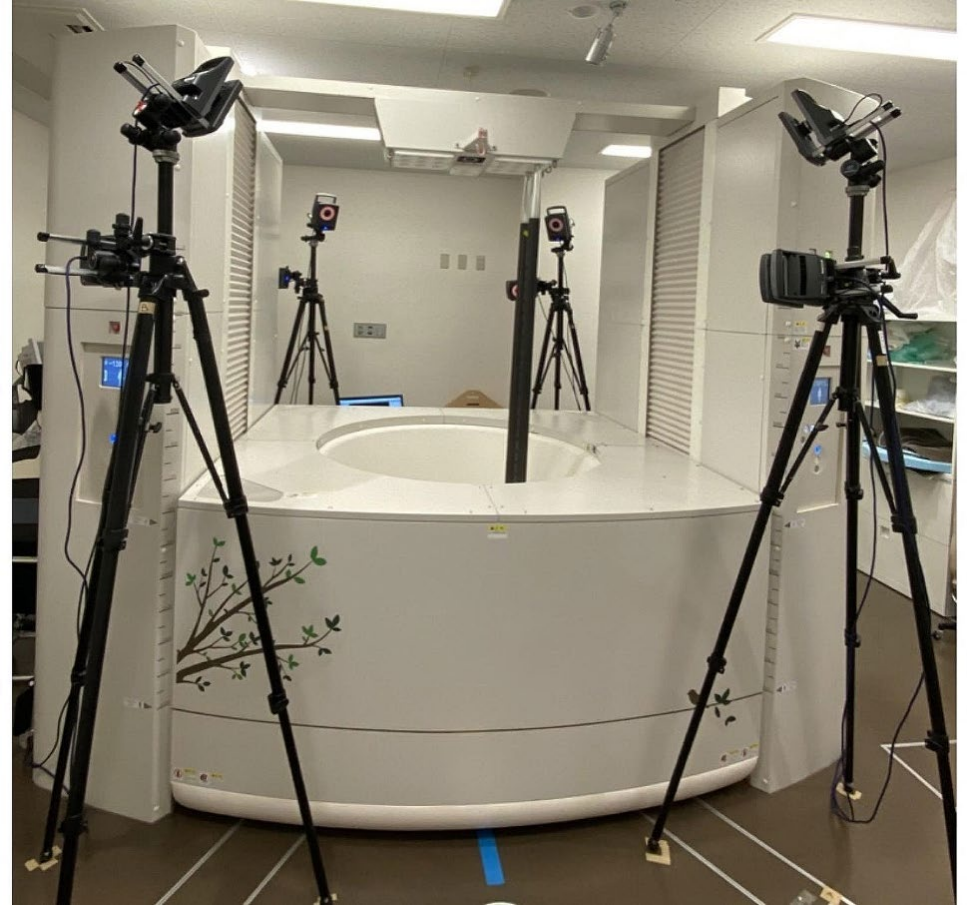
Upright computed tomography (CT) and optical motion capture cameras

Upright CT (prototype TSX-401R; Canon Medical Systems Corporation, Otawara, Japan) [12, 13] and an OMC system (Vicon MX-T series; Vicon Motion Systems, Oxford Metrics, Oxford, UK) (Fig. 1) were used to obtain shoulder kinematic data, as performed in our previous study [9]. In evaluating the AMC and SSMC methods, the Vicon Motion System was selected for capturing marker movement, demonstrating a high level of accuracy in marker tracking [14]. This system has consistently confirmed its reliability and is used for the validation of emerging motion capture devices in shoulder motion [15, 16].

### Procedures

**Fig. 2:**
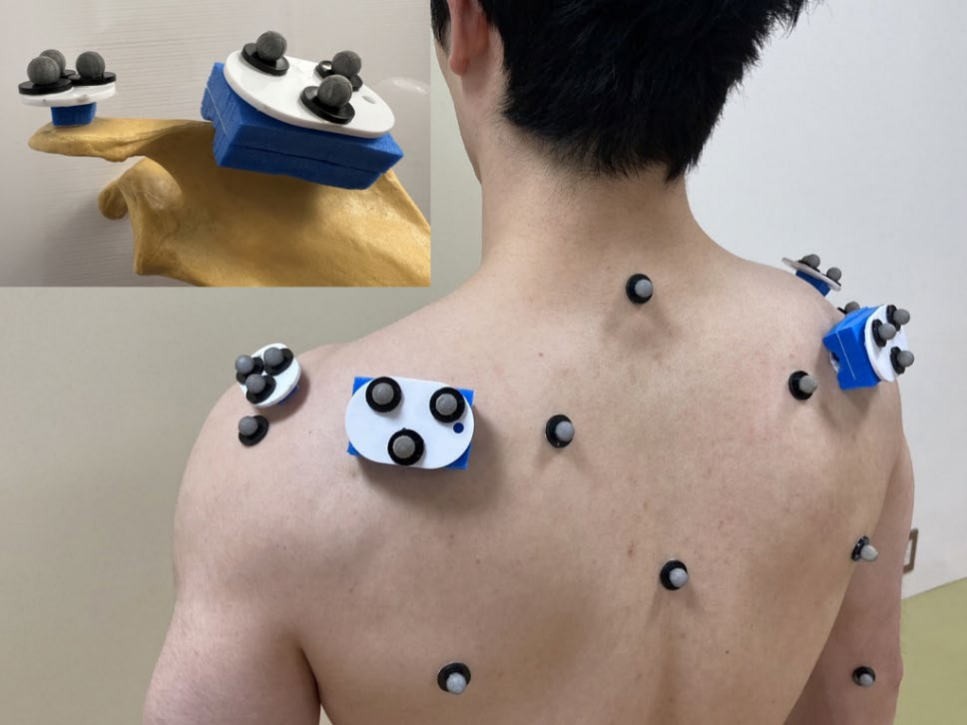
Spherical reflective skin markers were attached to the upper body according to the recommendation of the International Society of Biomechanics. Marker clusters were placed on the flat part of the acromion and the mid-portion of the scapular spine

Reflective skin markers were attached to the participants according to the recommendation of the International Society of Biomechanics (ISB) [17]. Marker clusters were placed on the flat part of the acromion and the mid-portion of the scapular spine (Fig. 2).

Participants received instructions to gradually raise both arms from the initial lower position to the highest point of elevation, all within a 10-second interval in the upright CT gantry. Considering the risk of OMC camera damage from radiation exposure, upright 4DCT and OMC were performed separately under the same conditions, with the participants’ elevating their hands along the poles set up in the upright CT gantry. They practiced this elevation, which is similar to a “hands up” motion, for at least three times using a timer before the shoulder motions were recorded.

In the upright 4DCT analysis, both static and dynamic CT images were acquired and accumulated in the Digital Imaging and Communications in Medicine (DICOM) data format. Static three-dimensional (3D) CT images were obtained in both reference and elevated positions. Complete bone data of the scapula, humerus, thorax, and seventh cervical to eighth thoracic vertebrae were acquired. Dynamic images during the active elevation were obtained via 4DCT for 10.275 s at 5 Hz with 0.5 mm-thick slices of CT volumetric data. Image reconstruction was performed using Adaptive Iterative Dose Reduction 3D, which could reduce imaging radiation [18]. In this study, the average dose‐length product and the effective dose estimate using a chest conversion factor of 0.014 were 660.4 mGy.cm and 9.2 mSv, respectively.

**Fig. 3:**
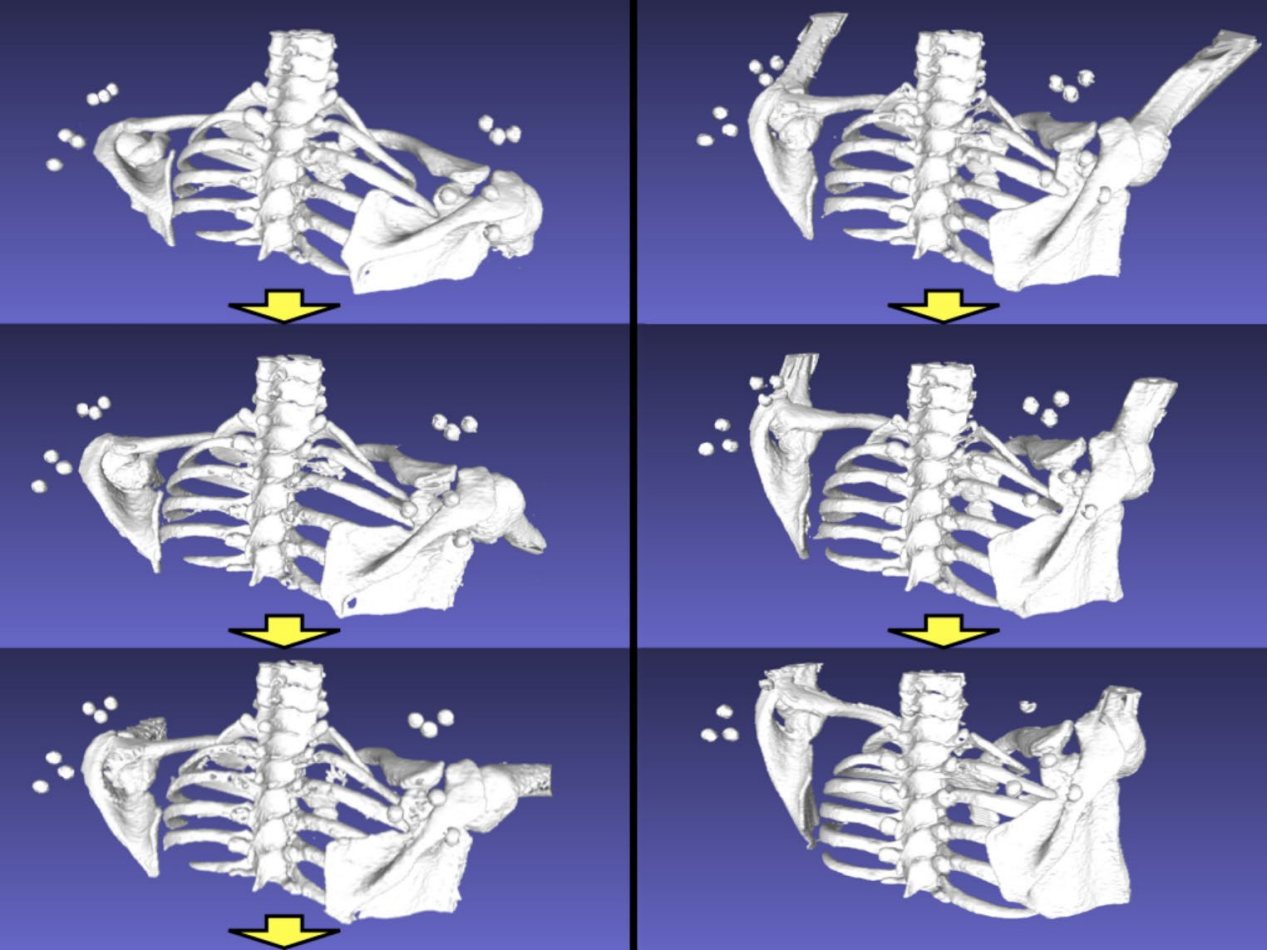
Four-dimensional CT images of the in vivo shoulder kinematics and marker clusters

Surface reconstruction was performed for both static and dynamic CT DICOM data using image segmentation software (Avizo Lite 9.3.0; Thermo Fisher Scientific) (Fig. 3). Regarding the 160 mm craniocaudal coverage of the 4DCT, surface registration was performed using a 3D-3D registration technique [19], which is an iterative surface registration with sequential volume CT DICOM data using Visualization Toolkit 8.1.2 (Kitware Inc.). Isolated surface data of the scapula, humerus, and thorax, obtained from static 3DCT, were registered to the 3D surface of all 4DCT frames using the iterative closest point (ICP) algorithm [19, 20]. The validation of the surface registration was carried out through perturbation analysis to evaluate its accuracy, and the results indicated a high level of reliability in previous studies [9, 19, 20].

In the OMC analysis, the CT gantry was lowered to its lowest position and eight OMC cameras were placed around it. The active shoulder elevation was recorded under the same conditions as in the upright 4DCT scan at a sampling frequency of 100 Hz. The scapular position was tracked with AMC and SSMC within the Vicon Nexus version 2.9 software and Vicon Bodybuilder version 3.6 software. Both marker cluster methods were single-calibrated in the reference position.

#### Rotation angles

Coordinate systems of the thorax, scapula, and humerus were defined according to the ISB recommendations [17]. Each bony landmark was identified in the static 3DCT images using the 3D mesh editing software (Meshlab 1.3.3; Institute of Information Science and Technologies, Pisa, Italy). After constructing the coordinate system, the kinematics of the object bone in relation to the reference bone were reconstructed using matrices representing the transformation from static 3DCT to each 4DCT frame [19]. Scapulothoracic, glenohumeral, and humerothoracic rotations were calculated using Euler angles with Y-X-Z, X-Z-Y, and Y-X-Y sequences, respectively. The scapulothoracic rotation angle was described as the upward/downward rotation about the X-axis, internal/external rotation about the Y‐axis, and anterior/posterior tilting about the Z‐axis. The glenohumeral rotation angle was described as the elevation/lowering about the X‐axis, internal/external rotation about the Y‐axis, and anterior/posterior plane of elevation about the Z‐axis. The humerothoracic elevation was described as the motion of the humerus relative to the thorax about the X‐axis. To synchronize the 4DCT and OMC data, the scapulothoracic and glenohumeral rotation angles were assessed for every 10º of humerothoracic elevation to approximate the closest two values with an assumption of linear change during the intervals of humerothoracic elevation.

#### Skin motion of the marker cluster

**Fig. 4:**
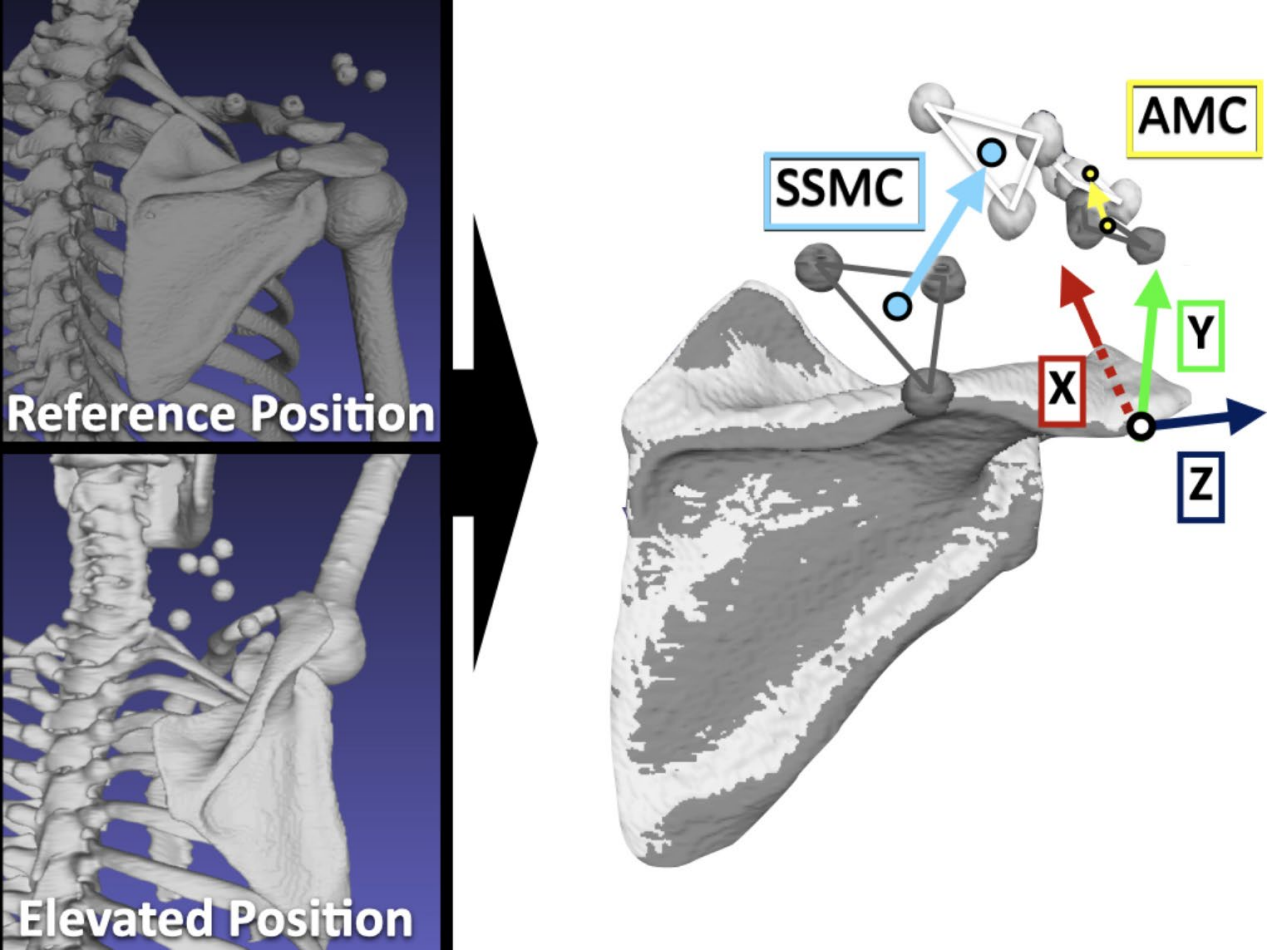
Three-dimensional (3D) surface models of scapular and marker clusters in the reference (*gray*) and elevated (*white*) positions. The 3D marker cluster movements were aligned with the scapular coordinate system. AMC, acromion marker cluster; SSMC, scapula spinal marker cluster

In the 4DCT images, the marker clusters were not always scanned because the craniocaudal coverage of the CT gantry was limited to 160 mm; thus, static 3DCT images of the reference and elevated positions were used to evaluate skin motion of the marker clusters. Marker cluster movements were calculated by matching the scapula in the reference and elevated positions. The marker cluster movement distances of AMC and SSMC from the reference position to the elevated position were calculated by center translation distances using Euclidean distance [5, 9]. The directions of these translations were also evaluated on the scapular coordinate system. The direction of the marker cluster translations was described as anterior/posterior movement for the X-axis translation, superior/inferior movement for the Y-axis translation, and lateral/medial movement for the Z-axis translation (Fig. 4).

### Statistical analysis

Statistical analyses were performed with SPSS version 29.0 (IBM Corp., Armonk, NY, USA).

The average values for the right and left shoulders were calculated for each participant. In the OMC analysis, measuring reliability of the AMC and SSMC methods were assessed by calculating the intraclass correlation coefficient (ICC). Reflective skin markers and marker clusters were attached three times to each of three participants by two of the authors, and rotation angles were subsequently calculated three times. The intra- and inter-observer reliabilities were assessed using ICC (1,3) and ICC (2,3), respectively. Two-way repeated-measures analysis of variance (ANOVA) was used to compare the scapulothoracic and glenohumeral rotation angles between the three scapular tracking methods (4DCT, AMC, and SSMC) and between humerothoracic elevation angles. Sidak post hoc tests were conducted for multiple pairwise comparisons when significant differences were noted in the ANOVA results. The differences in the marker cluster movement distance and directional translation between AMC and SSMC were evaluated using a paired t-test. The significance level was set as *P* < 0.05.

## Results

### Measuring reliability of the AMC and SSMC methods

The intra- and inter-observer correlation coefficients for AMC were 0.919 (95% confidence interval [CI], 0.411–0.998) and 0.923 (95% CI, 0.363–0.998) in scapulothoracic upward rotation, 0.972 (95% CI, 0.798–0.999) and 0.972 (95% CI, 0.798–0.999) in internal rotation, 0.878 (95% CI, 0.311–0.997) and 0.889 (95% CI, 0.385–0.997) in posterior tilting, 0.948 (95% CI, 0.627–0.999) and 0.949 (95% CI, 0.636–0.999) in glenohumeral elevation, 0.962 (95% CI, 0.730–0.999) and 0.963 (95% CI, 0.519–0.999) in external rotation, and 0.941 (95% CI, 0.574–0.998) and 0.941 (95% CI, 0.579–0.998) in anterior plane of elevation, respectively.

These coefficients for SSMC were 0.868 (95% CI, 0.432–0.997) and 0.879 (95% CI, 0.493–0.997) in scapulothoracic upward rotation, 0.945 (95% CI, 0.601–0.999) and 0.946 (95% CI, 0.616–0.999) in internal rotation, 0.795 (95% CI, 0.389–0.995) and 0.824 (95% CI, 0.433–0.995) in posterior tilting, 0.931 (95% CI, 0.504–0.998) and 0.933 (95% CI, 0.530–0.998) in glenohumeral elevation, 0.963 (95% CI, 0.737–0.999) and 0.964 (95% CI, 0.533–0.999) in external rotation, and 0.929 (95% CI, 0.489–0.998) and 0.931 (95% CI, 0.511–0.998) in anterior plane of elevation, respectively. All rotation angles calculated by both AMC and SSMC methods demonstrated high reproducibility.

### Scapulothoracic rotation

During active elevation, the scapulothoracic joint exhibited upward rotation, internal rotation, and posterior tilting. In 4DCT, the average upward rotation, internal rotation, and posterior tilting increased by 39.1° (from 3.4° to 42.5°), 6.9° (from 29.6° to 36.5°), and 21.1° (from − 4.2° to 16.9°), respectively, during 10°−140° of humerothoracic elevation.

In AMC, the average upward rotation, internal rotation, and posterior tilting increased by 26.9° (from 6.0° to 32.9°), 27.6° (from 28.3° to 55.9°), and 40.9° (from − 1.8° to 38.1°), respectively, during 10°−140°. of humerothoracic elevation. These findings reveal that the average differences between AMC and 4DCT were − 2.2° ± 7.5° in upward rotation, 14.0° ± 7.4° in internal rotation, and 6.5° ± 7.5° in posterior tilting.

In SSMC, the average upward rotation, internal rotation, and posterior tilting increased by 26.7° (from 6.7° to 33.4°), 7.4° (from 27.5° to 34.9°), and 24.5° (from − 2.0° to 22.5°), respectively, during 10 − 140° of humerothoracic elevation. The average differences between SSMC and 4DCT were − 7.5 ± 7.7° in upward rotation, 2.0 ± 7.0° in internal rotation, and 2.3 ± 7.2° in posterior tilting.

**Fig. 5:**
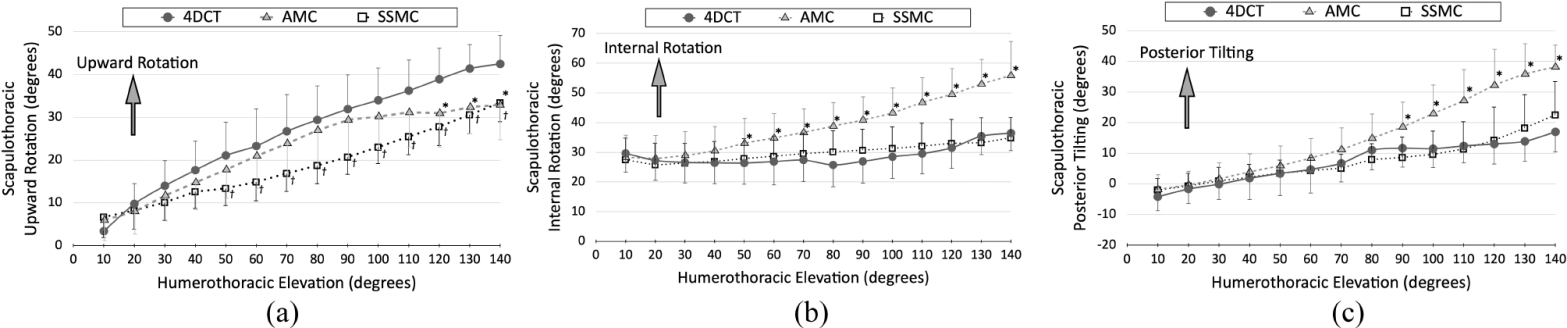
Scapulothoracic rotation angles tracked by upright four-dimensional computed tomography (4DCT), acromion marker cluster (AMC), and scapula spinal marker cluster (SSMC). The error bars show the standard deviation. *, significant difference between 4DCT and AMC; ^***†***^, significant difference between 4DCT and SSMC. *p* < 0.05

Significant interactions were observed between the scapular tracking methods and humerothoracic elevation angles for all scapulothoracic rotations (*p* ≤ 0.035). The main effects of the scapular tracking methods on humerothoracic elevation were assessed. The difference between AMC and 4DCT was significant at 120° of humerothoracic elevation in upward rotation (*p* = 0.015), 50° in internal rotation (*p* = 0.031), and 90° in posterior tilting (*p* = 0.047). In contrast, the difference between SSMC and 4DCT was significant at 50° of humerothoracic elevation in upward rotation (*p* = 0.015). No significant differences were observed in either internal rotation (*p* ≥ 0.403) or posterior tilting (*p* ≥ 0.086) (Fig. 5).

### Glenohumeral rotation

During active elevation, the glenohumeral joint exhibited elevation, external rotation, and anterior plane of elevation. In 4DCT, the average glenohumeral elevation, external rotation, and anterior plane of elevation increased by 51.0° (from 4.9° to 55.9°), 52.8° (from − 12.4° to 40.8°), and 85.4° (from 4.1° to 89.5°), respectively, during 10 − 140° of humerothoracic elevation.

In AMC, the average glenohumeral elevation, external rotation, and anterior plane of elevation increased by 63.1° (from 3.6° to 66.7°), 27.6° (from − 9.6° to 17.4°), and 40.9° (from 2.6° to 62.6°), respectively, during 10°−140° of humerothoracic elevation. Consequently, our findings revealed that the average differences between AMC and 4DCT were 3.7° ± 8.1° in glenohumeral elevation, − 8.3° ± 10.7° in external rotation, and − 8.6° ± 8.9° in anterior plane of elevation.

In SSMC, the average glenohumeral elevation, external rotation, and anterior plane of elevation increased by 58.3° (from 6.6° to 64.9°), 61.5° (from − 14.0° to 47.5°), and 91.4° (from 1.3° to 92.7°), during 10°−140° of humerothoracic elevation, demonstrating average differences of 8.8° ± 7.9° in glenohumeral elevation, 2.0° ± 9.1° in external rotation, and 1.9° ± 10.1° in anterior plane of elevation between SSMC and 4DCT.

**Fig. 6:**
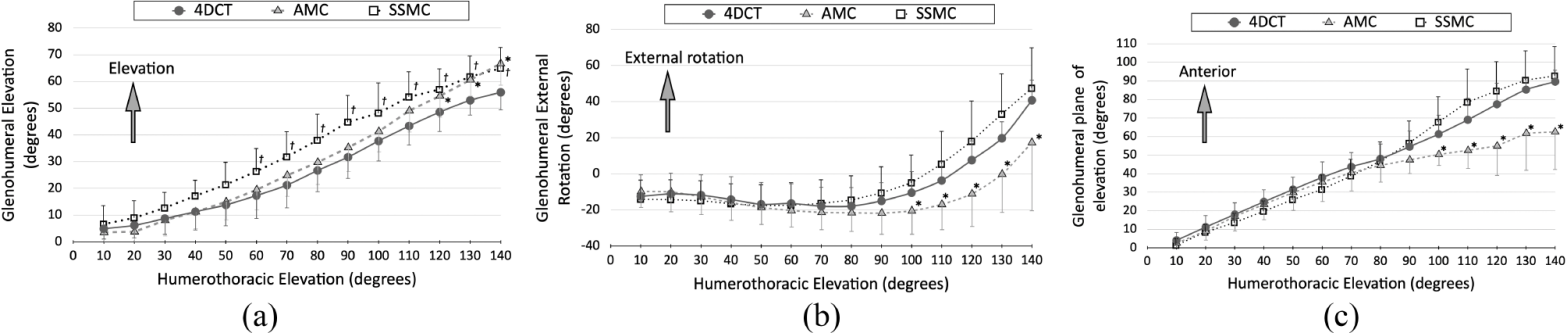
Glenohumeral rotation angles tracked by upright four-dimensional computed tomography (4DCT), acromion marker cluster (AMC), and scapula spinal marker cluster (SSMC). The error bars show the standard deviation. *, significant difference between 4DCT and AMC; ^***†***^, significant difference between 4DCT and SSMC. *p* < 0.05

Significant interactions were observed between the scapular tracking methods and humerothoracic elevation angles for all glenohumeral rotations (*p* ≤ 0.031). The simple main effects of the scapular tracking methods with humerothoracic elevation were assessed. The difference between AMC and 4DCT became significant at 120° of humerothoracic elevation in glenohumeral elevation (*p* = 0.026), 100° in external rotation (*p* = 0.024), and 100° in anterior plane of elevation (*p* = 0.016). In contrast, the difference between SSMC and 4DCT became significant at 60° of humerothoracic elevation in glenohumeral elevation (*p* = 0.048). No significant differences were observed in either external rotation (*p* ≥ 0.179) or anterior plane of elevation (*p* ≥ 0.197) (Fig. 6).

### Skin motion of the marker cluster

The average marker cluster movement distances were significantly smaller in AMC (28.7 ± 4.0 mm) than in SSMC (38.6 ± 5.8 mm) (*p* = 0.015). Both marker clusters were translated anteriorly (AMC, 15.9 ± 6.8 mm; SSMC, 18.3 ± 4.6 mm), superiorly (AMC, 16.3 ± 4.1 mm; SSMC, 30.9 ± 4.0 mm), and laterally (AMC, 14.3 ± 8.1 mm; SSMC, 13.2 ± 4.7 mm). There was a significant difference between AMC and SSMC movement in superior translation (*p* < 0.001). However, no significant differences were observed in the anterior and lateral translations (anterior translation, *p* = 0.300; lateral translation, *p* = 0.774).

## Discussion

This study evaluated the accuracy of the AMC and SSMC methods for tracking scapular motion during active shoulder elevation in comparison with upright 4DCT analysis. AMC showed a smaller difference from 4DCT in X-axis rotation angles (scapulothoracic upward rotation and glenohumeral elevation). Whereas, SSMC exhibited a smaller difference from 4DCT for Y-axis (scapulothoracic internal rotation and glenohumeral external rotation) and Z-axis (scapulothoracic posterior tilting and glenohumeral plane of elevation) rotation angles. Marker cluster movements were significantly smaller in AMC than in SSMC. Although scapular marker positional error during elevation is smaller for the acromion as noted in previous studies [4, 5], SSMC was considered more accurate than AMC in evaluating Y-axis and Z-axis rotation angles. Because the deltoid muscle is attached proximally to the acromion, the marker cluster attached to the acromion is more sensitive to deltoid muscle contraction than to scapular spine movements. Consequently, scapular STA might be influenced not only by positional errors but also by the inclination of the marker cluster. Consistent with previous validation studies, AMC indicated that X-axis rotation angles tended to exhibit greater accuracy compared to the Y- and Z-axis rotation angles [21–23]. One potential explanation for these results could be attributed to the inclination of the marker cluster. Therefore, it is essential to consider both positional errors resulting from skin motion and the impact of marker cluster inclination when using the marker cluster method.

The accuracy of AMC has been validated by comparisons with palpation [10, 11], scapula tracker [24, 25], or bone pins [26], and the errors were generally larger in the Y-axis rotation angles than in the other axes [3]. This finding is similar to our results where AMC showed the largest differences from 4DCT in the Y-axis rotation angles, particularly in scapulothoracic internal rotation. In contrast, SSMC showed no significant differences from 4DCT in the Y-axis rotation angles, implying the possibility of compensation for this error.

Similar to the comparison of AMC and SSMC accuracies in this study, a study compared the accuracy of AMC with that of a skin-fixed scapula tracker that was attached to the mid-portion of the scapula spine with an adjustable foot positioned on the acromion [25]. The scapula tracker was more accurate than AMC in the evaluation of Y-axis and Z-axis rotation angles as noted for SSMC in this study. Therefore, it might be better to place the marker cluster at the scapular spine in the evaluation of the Y-axis and Z-axis rotation angles. Although more accurate methods to evaluate scapular kinematics such as multibody kinematic optimization [27, 28] have been developed, the marker cluster methods calculating the X-axis rotation for AMC and Y-axis and Z-axis rotations for SSMC might be useful as one of the more convenient accurate methods of OMC.

The strength of this study was the dynamic validation of OMC to track movements of the shoulder bone using upright 4DCT. However, there are some limitations. First, the difference in the position of the marker cluster were only evaluated at two positions, and there might be more accurate AMC and SSMC attachment positions. Although we used the classical AMC method, which is a single calibration with the attachment of the marker cluster to the flat part of acromion [29, 30], AMC may be more accurate with multiple calibration [21, 25] or attachment to the marker cluster on the scapular spine side of the acromion [6]. Second, the present study solely assessed one specific movement due to the restriction of movement in the CT gantry. Different movements may provide different results. However, previous studies that investigated the errors in other elevation planes found similar error values to those obtained during elevation in this study [3, 25]. Third, very slow motion was evaluated to prevent motion artifacts. If we vary the speed of movements, we may obtain different outcomes. Fourth, this study included only young males with low average BMI; thus, our results may differ among females and individuals with different body types. More specifically, it is possible that individuals with greater subcutaneous tissue may exhibit different results, limiting the generalizability of these findings to the broader population.

## Conclusions

This study validated the accuracy of AMC and SSMC methods in comparison with upright 4DCT. In the scapulothoracic and glenohumeral rotations, AMC for X-axis rotation angles and SSMC for Y-axis and Z-axis rotation angles were considered more accurate as they showed smaller differences compared with 4DCT. It might be better to place the marker cluster in the scapular spine in the evaluation of Y-axis and Z-axis rotation angles. Our results will be useful in understanding and improving the accuracy of OMC.

## Data Availability

The datasets used and/or analyzed during the current study are available from the corresponding author on reasonable request.

## Abbreviations

AMC: Acromion marker cluster.
CI: Confidence interval.
CT: Computed tomography.
DICOM: Digital Imaging and Communications in Medicine.
ICC: Intraclass correlation coefficient.
ICP: Iterative closest point.
ISB: International Society of Biomechanics.
OMC: Optical motion capture.
SSMC: Scapula spinal marker cluster.
STA: Soft-tissue artifact.
3D: Three-dimensional.
4D: Four-dimensional.
